# Word Detection in Individual Subjects Is Difficult to Probe With Fast Periodic Visual Stimulation

**DOI:** 10.3389/fnins.2021.602798

**Published:** 2021-03-03

**Authors:** Lydia Barnes, Selene Petit, Nicholas A. Badcock, Christopher J. Whyte, Alexandra Woolgar

**Affiliations:** ^1^MRC Cognition and Brain Sciences Unit, University of Cambridge, Cambridge, United Kingdom; ^2^Department of Cognitive Science, Macquarie University, Sydney, NSW, Australia; ^3^Macquarie Centre for Reading, Macquarie University, Sydney, NSW, Australia; ^4^School of Psychological Science, University of Western Australia, Perth, WA, Australia

**Keywords:** reading, language, EEG, word detection, fast periodic visual stimulation, steady state evoked response

## Abstract

Measuring cognition in single subjects presents unique challenges. On the other hand, individually sensitive measurements offer extraordinary opportunities, from informing theoretical models to enabling truly individualised clinical assessment. Here, we test the robustness of fast, periodic, and visual stimulation (FPVS), an emerging method proposed to elicit detectable responses to written words in the electroencephalogram (EEG) of individual subjects. The method is non-invasive, passive, and requires only a few minutes of testing, making it a potentially powerful tool to test comprehension in those who do not speak or who struggle with long testing procedures. In an initial study, Lochy et al. (2015) used FPVS to detect word processing in eight out of 10 fluent French readers. Here, we attempted to replicate their study in a new sample of 10 fluent English readers. Participants viewed rapid streams of pseudo-words with words embedded at regular intervals, while we recorded their EEG. Based on Lochy et al. (2015) we expected that words would elicit a steady-state response at the word-presentation frequency (2 Hz) over parieto-occipital electrode sites. However, across 40 datasets (10 participants, two conditions, and two regions of interest–ROIs), only four datasets met the criteria for a unique response to words. This corresponds to a 10% detection rate. We conclude that FPVS should be developed further before it can serve as an individually-sensitive measure of written word processing.

## Introduction

Measures of cognitive processes that are sensitive to individual-level effects can be powerful research tools. Knowledge about individual variance can inform cognitive models and data about individual cognition can have important clinical application. For example, in cognitive neuropsychology, a double dissociation between an individual who could read familiar words but struggled to sound out new words, and an individual who could sound out new words but struggled to read familiar words, gave rise both to the influential Dual Route Cascaded model of reading (Coltheart et al., 2001) and to targeted interventions for distinct reading problems. However, drawing conclusions from individual subjects’ data is often difficult. The influence of measurement error and other sources of “noise” in the data is high in individuals relative to groups, and in clinical applications we often cannot mitigate the noisy single-subject data by testing for long periods.

An emerging method, called “fast, periodic, and visual stimulation,” or FPVS, is proposed to provide a solution to some of these challenges. It is designed to be a fast way to test cognitive processes in individual subjects with high signal-to-noise ratio (Rossion et al., 2015). In FPVS, participants view rapidly presented stimuli while their neural responses are recorded with electroencephalography (EEG). The stimuli are presented periodically at a certain predictable frequency. Within this stream, we can present stimuli belonging to different categories at different periodic frequencies. For example, we may present scrambled face images every 100 ms (that is, at 10 Hz), with every fifth face presented unscrambled (i.e., unscrambled faces presented every 500 ms, at 2 Hz). The periodic stimulation is designed to elicit an oscillatory response in the EEG signal at the presentation frequency, in this case at 10 Hz. If the brain also differentiates the embedded or “oddball” category—in this case, the unscrambled faces—that category should also elicit an increased response at the embedded frequency of 2 Hz. Several studies have demonstrated robust responses to the embedded category such as for faces among scrambled face images, for faces among non-face objects, and for new face identities among repeating identities (Rossion, 2014; Rossion et al., 2015). Because the stimulus stream is presented quickly, we can present many stimuli in a few minutes, keeping testing short (approximately 3 min). The high signal relative to noise associated with the large number of stimulus presentations is proposed to support individual-level tests of cognitive processes (Liu-Shuang et al., 2014).

The success of these initial face processing studies with FPVS could partly rest on human’s unique face recognition expertise. Recently, researchers have begun to examine whether the same approach can be used to test word recognition. Like face processing, recognising words is a key step in our social and intellectual development. Visual word recognition and face processing are commonly associated with parallel hubs in the left and right fusiform gyri respectively, giving rise to the hypothesis that the visual expertise underlying both processes rests on related mechanisms (McCandliss et al., 2003). In the EEG time course, visual words elicit a larger negative deflection relative to unfamiliar scripts around 170 ms, while face images elicit an analogous “N170” response relative to non-face objects (Bentin et al., 1999). These similarities make word recognition an ideal way to test whether FPVS can be useful in other cognitive domains. On the other hand, evidence that word responses at 170 ms represent any extraction of word meaning is inconsistent, with some studies finding that we can differentiate familiar and unfamiliar scripts, but not the content of words, at that time in the EEG signal (Bentin et al., 1999). Core processing for word meaning appears to emerge around 400 ms from stimulus onset (Kutas and Federmeier, 2011). Further, the point at which we can extract a word’s meaning depends on the word’s length and how frequently we encounter it (Hudson and Bergman, 1985). Thus, stimulus onset for words may be a poorer marker of word processing onset than stimulus onset for faces. Translating FPVS for face processing to word recognition could present some unique challenges.

A study by Lochy et al. (2015) shows promising individual-level effects of word processing using FPVS. They presented words embedded in a stream of scrambled fonts (unfamiliar scripts), non-words (unpronounceable letter strings), or pseudo-words (pronounceable but meaningless letter strings). Words appeared at 2 Hz in a 10 Hz stream. Words elicited a unique, or “oddball,” response relative to scrambled fonts in 10 out of 10 participants, and relative to non-words and pseudo-words in eight out of 10 participants, in under 5 min testing for each condition.

These results are exciting because they suggest that FPVS can robustly measure the neural correlates of word recognition on an individual subject level, with minimal testing time. However, individual-level sensitivity could be lower than these headline results suggest. First, fewer people showed an oddball response for words among pseudo-words (8/10), compared to words among scrambled fonts (10/10). This word response among pseudo-words is especially important. Whereas words and scrambled fonts differ in their constituent objects, words and pseudo-words differ in whether the letters form a word. Thus, we can more confidently infer that a person recognises words by looking at their response to words among pseudo-words. Second, this high instance of individual-level word/pseudo-word effects was only reported for the first experiment. In this experiment, neighbouring letters in pseudo-words were not matched to the word stimuli. Particular letter combinations, or “bigrams,” are more common than others. For example, in English, “st” is common, while “pd” is rare. Because the first experiment did not match bigram frequency between the words and pseudo-words, the oddball response may have reflected people’s familiarity with letter sequences, as well as, or instead of, recognition of the specific word. The authors tested for a word-specific response among bigram-frequency-matched pseudo-words in a follow-up experiment. They reported a similar group effect but did not report the individual level detection rate, that is, whether the response at the oddball frequency was greater than noise for each participant.

If FPVS can be used to reliably track the neural correlates of word processing in individual subjects, it could have important research and clinical applications. Therefore, we sought to replicate the effect of Lochy et al. (2015), and report the individual-subject detection rate for stimuli matched on bigram frequency. We first established the validity of our implementation by testing whether we could reliably detect faces among scrambled faces. This effect has been replicated many times, so we used it as a sanity check for whether our stimulus presentation and analyses were appropriate. Then, for word processing, we used the design reported in Lochy et al. (2015) that produced a high rate of significant effects at the individual-subject level. We used stimulus delivery routines provided by the Rossion group, and the same analysis software and pipeline as that described by Lochy et al. (2015), but used our own stimuli (in English, the original study was in French), participants, and EEG recording system. We asked whether we could replicate their fast, individually-sensitive effects reflecting differential processing of words and pseudo-words. Surprisingly, despite strong individual-subject responses for faces, we found detectable individual-level differentiation of words and pseudo-words in only four out of 40 datasets, suggesting that the method may not be as robust as the initial data suggested.

## Materials and Methods

### Participants

We recruited 10 neurotypical adults (seven female, three male, mean age 19.6 ± 1.5) from undergraduate and paid participant pools at Macquarie University. As the study was designed to assess the method at the level of individual subjects, the critical feature for statistical power was the number of trials, rather than the number of participants, so we chose *N* = 10 to match Lochy et al. (2015). Participants were informed of the aims of the study and gave written consent before taking part. The study was approved by the Macquarie University Human Research Ethics Committee (approval no. 5201200658).

### Stimuli

#### Faces

We used the 50 face images, and 50 scrambled face images from Rossion et al. (2015) (available at^1^). The 50 scrambled images had been made by randomly repositioning the pixels in each face image. Consequently, the scrambled faces did not contain coherent object-like features, but had some of the low-level features of faces such as luminance and contrast. It was important for us that the face and non-face stimuli were clearly distinct, as we included this condition to test whether our methods could detect noticeably different stimuli. Because each face image produced a single scrambled face image, scrambled face images repeated four times as often as face images to produce the required ratio. To account for differences in screen resolution between our computer monitor and that used by Lochy et al. (2015), we presented the face and scrambled-face stimuli at twice their original pixel height and width (from 200 × 200 to 400 × 400 pixels), and jittered them between 88 and 112% of their mean size. When viewed from 1 m away, the images subtended 6.28–8.03 degrees of visual angle in either plane (compared to 5.22 degrees in Rossion et al., 2015).

#### Words (Original)

For the word condition, we used 24 common English words and 96 pronounceable pseudo-words (Supplementary Table 1). Each stimulus was four letters long. We chose to use four-letter words, rather than five-letter words as in Lochy et al. (2015), to maximise the likelihood that the words were acquired early and frequently used. This would make the stimulus set appropriate for fluent readers, and for future use with less fluent readers (e.g., children) or people whose reading ability is unknown. We first chose the 3,000 most imageable words from the Cortese imageability database (Cortese and Fugett, 2004). We entered these words into the Medical College of Wisconsin’s MCWord database to extract word statistics. We sorted the words by most frequently produced, then by imageability. We then used the Oxford Word List, which records words used in Australian children’s writing (^2^ retrieved 15 May 2020), to identify the most frequently written words in Year 2 (typically at age 7–8). This gave us a complementary frequency measure defined by children’s natural word generation. We matched these words with our Cortese word list and selected the 24 items best matched for frequency, imageability, and Year 2 production. We rejected words that were plural (for example, “days”) or both a noun and a verb (for example, “left”).

We built each pseudo-word by changing the first letter of each target word to another letter that made a pronounceable pseudo-word. We rejected imageable words that could not form appropriate pseudo-words (for example, “play”). We replaced these with the next item on our Cortese-Oxford-matched word list. We created four pseudo-words for each word so that each item would appear equally often in our word stream. This should increase experimental control compared to Lochy et al. (2015), as word-specific responses cannot reflect how often they appear in the stream. We submitted each pseudo-word to MCWord to extract word statistics. If a candidate pseudo-word produced a non-zero frequency rating (i.e., was actually a word), we changed its first letter until the frequency rating was zero. We then calculated the bigram frequency mean and standard deviation for each of the five word lists (one word list and four pseudo-word lists). We used a *t*-test to confirm that there was no evidence for a reliable difference in bigram frequency between each pseudo-word list and the word list (all *ps* > 0.05, all mean bigram frequencies between 26 and 28). Finally, we used a MATLAB script (MATLAB R2012b, 2012) to generate a JPEG file of each item.

Words and pseudo-words were presented in black Verdana font on a grey background, covering between 1.93 and 6.02 degrees (compared to Lochy et al.’s 3.7–6.7 degrees) of the visual field horizontally and between 1.01 and 2.05 degrees (compared to Lochy et al.’s 1.0–1.8 degrees) of the visual field vertically when viewed from 1 m with a screen resolution of 1,920 × 1,080 pixels.

#### Words (Large)

Due to different word choices, our words were on average narrower than those in Lochy et al. (2015). To account for the possibility that larger stimuli may be important to elicit clear effects, we also generated large word and pseudo-word stimulus sets by doubling the width and height of our original stimuli. Large word stimuli covered between 3.86 and 12.04 degrees of the visual field horizontally and between 2.02 and 4.1 degrees of the visual field vertically when viewed from 1 m with a screen resolution of 1,920 × 1,080 pixels.

### Procedure

#### EEG Procedure

Data were recorded with a 64-channel BioSemi system sampling at 2,048 Hz, using ActiView705 v8.6.1. We first fitted participants with an elastic BioSemi cap and cleaned sites for electromyography with alcohol wipes. We then placed electrodes on the left and right mastoid process, below the right eye, and at the outer canthus of the right eye. Scalp EEG electrodes were attached to the cap in International 10/20 system layout, and connected to the scalp with Signa electrolytic gel. Electrode offsets were below 50 mV.

#### Task

**FIGURE 1:**
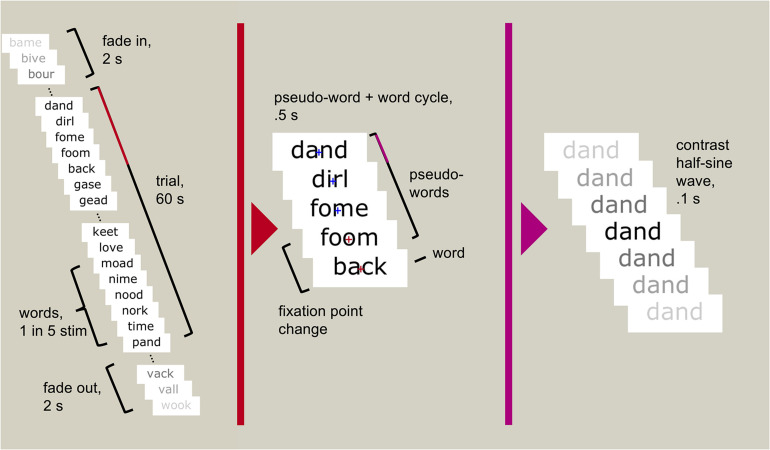
An example trial sequence using words and pseudo-words. Each stimulus reached maximum contrast and returned to minimum contrast within 100 ms. Every fifth stimulus was a real word (every 500 ms, or 2 Hz), the rest were pseudo-words. Participants were asked to attend to the fixation cross (depicted only in the middle panel but always visible to the participant) and press a button when it changed colour (randomly occurring between six and eight times throughout the 60 s trial).

Participants sat 1 m from a computer screen. The task was presented on a 27-inch Samsung S27A950 LED monitor using MATLAB 2012b v8.0.0.783 32-bit (MATLAB R2012b, 2012) and Psychtoolbox v3.0.10 (Brainard, 1997; Pelli, 1997; Kleiner et al., 2007). We used presentation scripts provided by the Rossion group which used the same sinusoidal contrast modulation function used by Lochy et al. (2015) (SinStim v1.8.9) to fade each stimulus in and out. A fixation cross appeared centrally to mark the start of the trial. The trial began with a random foreperiod between 2 and 5 s, followed by the stimulus stream (Figure 1). Each stimulus began at 0% contrast, gradually increased to a maximum value, and returned to 0% within 100 ms, creating a stream of stimuli reaching maximum contrast 10 times per second (at 10 Hz). The core of the stream was 60 s full contrast modulation, in which the maximum contrast was 100% (that is, black stimuli on the white background). The 60 s period was flanked by two-second fade-in and fade-off periods, in which the maximum contrast gradually increased from 0 to 100%, or decreased from 100 to 0%. In each condition, every fifth stimulus was categorically different to the other stimuli; i.e., one face followed four scrambled faces, or one word followed four pseudo-words. This created a 2 Hz stimulation rate for the “oddball” stimuli, faces, and words. With 120 unique stimuli presented pseudorandomly to minimise repetitions, this entailed five repetitions of each stimulus within the 60 s of high contrast stimulation.

The fixation cross remained centred on screen through the trial. Between six and eight times during the trial, at pseudorandomly-spaced intervals, the fixation cross changed from blue to red for 400 ms. Participants were asked to respond to the change in colour by pressing the spacebar on a computer keyboard. The task was designed to ensure that participants maintained focus on the screen without requiring them to engage with the oscillating stimuli.

Participants completed three one-minute trials of each condition: faces and scrambled faces, words and pseudo-words, and large-sized words and large-sized pseudo-words. Condition order was pseudorandomised across participants. We also tested two other conditions for a related project. Testing time was approximately 20 min.

### Analyses

#### Pre-processing

We followed the analysis steps of Lochy et al. (2015). First, we imported data into Letswave 6^3^, a MATLAB toolbox used by Rossion et al. (2015) and Lochy et al. (2015) to extract the signal-to-noise ratios of specific frequencies from EEG data. Within this toolbox we processed the Biosemi data with a fast Fourier transform (FFT) filter, width 0.01–1 and cut-off 0.1–100. We segmented the trials to include fade-in and fade-out periods, creating a 64 s epoch. We then downsampled the data to 250 Hz, linearly interpolated the signal, and re-referenced to the common average. Next, we re-segmented the trials, this time to include only the period of full-contrast stimulation. Because of slight inaccuracies in the monitor refresh rate, stimuli were presented at 10.0007 Hz. Thus, the maximum number of oddball cycles in our 60 s trial was completed at 59.996 s. Following Lochy et al. (2015, 2016), we segmented the trials to this more precise duration. We refer to the stimulation frequency as 10 Hz for simplicity.

Each subject completed three trials in each condition. First, we averaged the data from the three segmented trials in each condition together to form a single grand average trial, lasting 59.996 s, for each condition. Then, we transformed the grand average data into the frequency domain using a FFT. We took the absolute value of the FFT, retaining the units of the original data, amplitude in microVolts. We normalized the amplitude by the length of the trial (200 Hz × 60 s). We then calculated a ratio between the amplitude of response at each frequency and the amplitude of response at neighbouring frequencies, which we refer to as the signal-to-noise ratio (SNR) of each frequency, in keeping with previous FPVS work (Lochy et al., 2015, 2016; Rossion et al., 2015). For this, we followed the method described in Rossion et al. (2015) and implemented in Lochy et al. (2015, 2016). For each frequency, we defined the SNR as the amplitude at the frequency divided by the average amplitude of 19 neighbouring bins. These bins comprised 10 bins on each side of the frequency of interest, each 0.0166 Hz wide, omitting immediate neighbours to the frequency of interest, and the average was computed using the values from all but the bin with the highest amplitude, following Lochy et al. (2015). Separately, we calculated *z*-scores for each frequency as the difference in amplitude between that frequency and the mean of 19 neighbouring bins (selected in the same way as for SNR), divided by the standard deviation of the neighbouring bins.

#### Regions of Interest

We expected the base stimulation rate to drive a visual response, so we selected Oz as the electrode of interest. For word and face oddballs (at 2 Hz), we selected symmetrical left- and right-hemisphere regions of interest (ROIs). These were centred on PO7 or PO8 and included the four closest electrode sites (P5, P7, PO3, O1; and P6, P8, PO4, and O2). These scalp locations produced the strongest overall signal-to-noise ratios for the contrast of words and pseudo-words in Lochy et al. (2015). We used their electrode sites *a priori*, rather than focusing on electrode sites with the strongest signal-to-noise ratio (as in Lochy et al., 2015) to avoid possible circularity in the analysis (Kriegeskorte et al., 2009; Kilner, 2013). These sites are also approximately above the fusiform gyrus, which is strongly implicated in both face and visual word processing (e.g., Behrmann and Plaut, 2014). We considered data from left- and right-hemisphere ROIs separately, as averaging across hemispheres could obscure a lateralised effect: faces commonly elicit stronger responses from the right fusiform gyrus, while words typically elicit stronger responses from the left (Rossion et al., 2003; Maurer et al., 2008), which could emerge in our left and right ROIs. However, this lateralisation can vary from person to person, so we did not make specific predictions about where face or word effects should surface for each participant. Following Lochy et al. (2015) and in order to be as liberal as possible in replicating the original effect, we did not correct for multiple comparisons across the two ROIs in each oddball analysis. However, assessing two ROIs increases our chance of incorrectly rejecting the null hypothesis (i.e., inflates the false positive rate), so the true effects are likely to be even weaker than we report here (see Supplementary Material 2 for a deconstruction of our analysis over conditions and ROIs).

#### Statistical Analyses

##### Group Effects

Although our main interest was in individual-subject sensitivity, we first ran a group-level analysis to compare directly with Lochy et al.’s group word-pseudo-word differentiation, and to identify any weak effects that may not appear at the individual level. For each participant, we extracted signal-to-noise ratios at the base rate of 10 Hz, the oddball stimulation rate and its harmonics, 2, 4, and 6 Hz. The harmonic at 8 Hz was excluded, following Lochy et al. (2015), who reported that group-level *z*-scores were not reliably greater than noise at 8 Hz across all conditions. Next, signal-to-noise ratios were averaged across the harmonics, creating two oddball signal-to-noise ratios (left and right ROIs) for each participant in each condition. We collated all participants’ values for each condition and compared them to one (the noise level) using Wilcoxon’s signed rank test implemented through the statistical package JASP (Love et al., 2015; compared to the parametric one-tailed *t*-test in Lochy et al.).

##### Individual Effects

For individual-level analysis, we extracted data from Oz at the base stimulation rate (10 Hz), and data averaged across each ROI at the oddball stimulation rate (now the average of 2, 4, and 6 Hz). For each frequency of interest we extracted *z*-scores at that frequency and the surrounding 20 bins, excluding the nearest neighbour on either side and removing the highest bin. This created two sets of 20 bins per participant per condition: one bin of interest, and 19 bins that in theory should contain no signal, separately for the base rate and for the oddball rate. In Letswave 6, we ranked the bins by their *z*-score. Following the logic of Lochy et al. (2015) if there was no effect (similar response in all bins), each bin would have an equal 5% chance of ranking first. Thus, allowing for a 5% false positive rate, we concluded that any participants whose strongest *z*-score was at the oddball frequency showed an oddball-specific response.

## Results

### Behaviour

All participants responded to fixation point changes with a hit rate of 70% or higher on each trial.

### Visual Steady State Response (10 Hz) at Oz

We first checked for responses driven by visual stimulation at the base frequency of 10 Hz, in the occipito-central electrode (Oz).

#### Group Analysis

The base stimulation frequency was reliably greater than noise in the face condition and both word conditions (Table 1). In the face condition, the median SNR at the base stimulation frequency was 22, reflecting an amplitude of response at 10 Hz that was 22 times the mean of the neighbouring frequencies. However, the standard deviation was also high (18 for the face condition), indicating substantial variation between participants. Word condition SNR at 10 Hz over Oz similarly showed both a large median and standard deviation, indicating that this basic visual response varies widely in size between participants.

**TABLE 1:** Descriptive and inferential statistics for visual steady state responses (SNR) at 10 Hz, reflecting the base stimulation frequency (visual stimulation).

	Median	SD	Wilcoxon’s V	*p*
Faces in scrambled faces	22.10	18.03	55.00	<0.01
Words in pseudo-words, original	8.01	12.70	55.00	<0.01
Words in pseudo-words, large	8.57	10.82	55.00	<0.01

*Wilcoxon’s V indicates the sum of the positive differences between observed and test values. Large values, relative to the number of observations, indicate a larger difference. Degrees of freedom = 9. Hypothesis: median signal-to-noise ratio is greater than the noise level, 1.*

#### Individual-Level Analysis

The base stimulation frequency also produced detectable individual-level effects (*p* < 0.05), as defined by Lochy et al. (2015) and described above, at Oz. This was the case for every participant in every condition, with two exceptions. One participant did not meet the criteria for a detectable effect in the word condition with large stimuli, but did in the original-sized word condition. Another participant showed the opposite pattern. All participants met our criteria for a visual steady-state response in at least one condition. We include individual-level frequency spectra for each condition in Supplementary Material 3. Note that, as suggested by the large standard deviations at the group level (Table 1), the strength of the effect varies substantially across individuals, despite a high statistical detection rate at the individual subject level.

### Oddball (Category) Response for Faces (2 Hz)

Next, we checked whether we could detect 2 Hz oscillatory responses reflecting the presentation of faces among scrambled faces, in our left and right ROIs centred on P07 and P08.

#### Group Analysis

**FIGURE 2:**
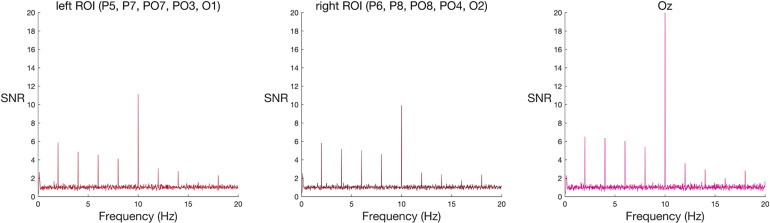
Frequency spectra for faces among scrambled faces (group-level results) at the two ROIs and Oz. Signal-to-noise (SNR) units represent the ratio of the amplitude at each frequency to the mean amplitude of 20 neighbouring frequencies. Large SNR values indicate a greater response at that frequency relative to nearby parts of the frequency band. A new face or scrambled face image appeared every 100 ms (10 Hz), eliciting visual steady state responses at 10 Hz in both left and right ROIs, and at Oz. This response is strongest at the peri-occipital electrode, Oz. Faces appeared every 500 ms (2 Hz), eliciting a spike in signal relative to noise at 2 Hz and its harmonics (4 Hz, 6 Hz, and 8 Hz) in both left and right ROIs, and at Oz.

As expected, oddball faces elicited a statistically reliable effect at 2 Hz in both the left and right ROI, as measured by Wilcoxon’s signed rank test (Table 2 and see Figure 2 for frequency spectra).

**TABLE 2:** Descriptive and inferential statistics for stimulus differentiation (SNR) at 2 Hz (oddball frequency).

		Median	SD	Wilcoxon’s V	*p*
Faces in scrambled faces	Left ROI	3.88	3.15	55.00	<0.01
	Right ROI	4.35	3.82	55.00	<0.01
Words in pseudo-words, original	Left ROI	1.05	0.34	29.00	0.46
	Right ROI	1.01	0.28	26.00	0.58
Words in pseudo-words, large	Left ROI	1.22	0.35	41.00	0.19
	Right ROI	1.08	1.59	32.00	0.70

*Degrees of freedom = 9. Hypothesis: median signal-to-noise ratio is greater than the noise level, 1.*

#### Individual-Level Analysis

In line with findings across the group, most (9/10) participants met the criteria for a face-specific response. These nine participants showed the effect in both ROIs. The remaining participant’s strongest signal-to-noise ratio in the sampled range did not fall on the face frequency in either the left or the right ROI.

### Oddball (Category) Response to Words (2 Hz)

Finally, we asked whether we could replicate the main result of interest: a 2 Hz oscillatory response reflecting the presentation of words among pseudowords.

#### Group Analysis

**FIGURE 3:**
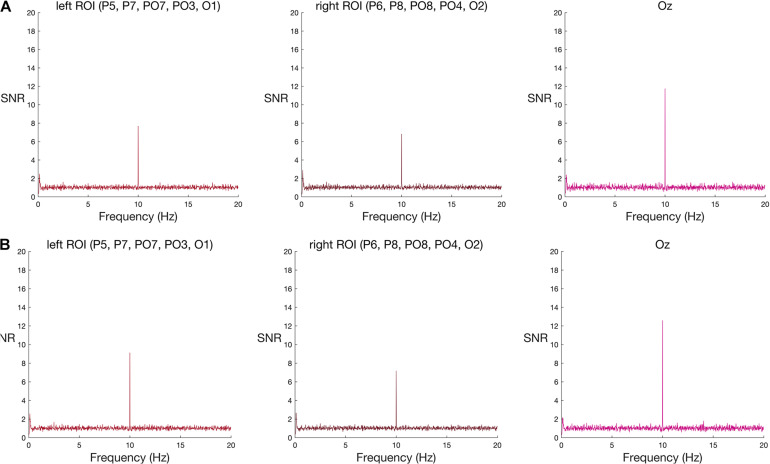
Frequency spectra for **(A)** original-sized and **(B)** large words among pseudo-words (group-level results) at the two ROIs and Oz. Signal-to-noise (SNR) units represent the ratio of the amplitude at each frequency to the mean amplitude of 20 neighbouring frequencies. A new word or pseudo-word appeared every 100 ms (10 Hz), eliciting a visual steady state responses at 10 Hz in both left and right ROIs, and at Oz. As with face stimuli, this base stimulation response was strongest at Oz. Words appeared every 500 ms (2 Hz). Word-specific responses were expected at 2 Hz and its harmonics (4 Hz, 6 Hz, and 8 Hz), but neither word condition elicited a group-level increase in amplitude at these frequencies, relative to neighbouring frequencies, in either ROI nor Oz. See Supplementary Material 3 for the corresponding individual-subject frequency spectra.

In contrast to the robust face effect, and counter to the outcome of Lochy et al. (2015), neither set of words reliably elicited a response greater than noise at the group level (Table 2 and see Figure 3 for frequency spectra).

#### Individual-Level Analysis

Again, in contrast to the face effect and the results of Lochy et al. (2015), but consistent with our failure to find significant effects at the group-level, individual word-specific responses were rare. For original-size stimuli, only one participant showed a word-specific response and only in the left ROI. For large stimuli, two participants showed a word-specific response; one in both ROIs, and the other only in the left ROI. In total, across four tests—two word-stimulus sizes and two ROIs—and 10 participants (a total of 40 datasets) only four datasets met the criteria for a unique response to words nested among pseudo-words, using an uncorrected alpha level of 5%. This corresponds to a 10% detection rate (see Supplementary Figure 3). When we corrected the alpha level, to control for the four multiple comparisons inherent in testing two ROIs and two stimulus sizes in each person, none of the datasets showed a significant effect (see Supplementary Table 2).

## Discussion

We set out to replicate the claim that fast, periodic stimulation could provide an individually-sensitive, minimally-demanding test of word recognition. Previous work suggests that FPVS can rapidly elicit a unique response to words embedded among pseudo-words in eight out of 10 people (Lochy et al., 2015). We asked whether word detection rates would be similarly high in a novel sample of English speaking adults with word and pseudo-word lists matched for bigram frequency. We expected to observe similarly high word-detection rates in adults who we knew were able to read, with the eventual aim of using the test for clinical purposes in the future.

Contrary to our prediction and the previous literature, we observed very low detection rates for neural entrainment to words embedded in pseudo-words. After validating our stimulus delivery and analysis pipeline using face stimuli (near perfect detection rate–18/20 datasets) we tested word detection in two ROIs (as in Lochy et al., 2015) with two stimulus sizes. Of the 40 word datasets, only four showed a statistically reliable effect, corresponding to just a 10% detection rate, even when using an uncorrected alpha of 5%.

One explanation for the disparity in detection rates could be that the limited sample size in both studies did not fully capture the distribution of responses. We limited our sample size to that of Lochy et al. (2015), anticipating that most participants would show a word-specific response. Our poor single-subject detection rate is sufficient to conclude that word detection with FPVS is not always as robust as one might anticipate given Lochy et al.’s 80% success rate, but leaves us unsure as to how much random variation exists across individuals in their neural responses to words. Nonetheless, it appears that FPVS may only elicit word-specific responses in a subset people, which would fundamentally limit how we use the method to study word processing in single subjects.

Another possibility is that we missed word-specific responses by focusing on specific ROIs. Since our aim was to replicate the work of Lochy et al. (2015), we based our ROIs on the peak responses reported in their work. Our ROIs were deliberately broad (each comprising five electrodes out of 64), reaching from O1 to P7, so as to be robust to individual variation in anatomy. This approach avoids the circularity associated with selecting the most responsive electrode for analysis (Kilner, 2013), but means it is possible that we missed responses present at other sensors. This limitation could be exacerbated by the fact that some brain regions that are implicated in reading, such as the occipito-temporal sulcus (Cachia et al., 2018), are difficult to record from with EEG. Our poor detection rate cannot be taken as evidence for an absence of word-specific responses in these participants, but is evidence that the FPVS method does not always detect them.

From a broader perspective, it is perhaps not surprising that a method developed for detecting differential responses to faces and non-face images does not elicit similarly stable effects for words among pseudo-words. The FPVS approach uses rapid stimulus presentations and analyses focused on induced oscillatory responses that are matched to the stimulus or oddball presentation frequency. Because of this, it is most sensitive to detecting neural responses that (a) occur despite limited stimulus presentation time, and (b) occur with sufficiently consistent latency and short duration, over trials, that they can be detected as an oscillatory response. We consider these requirements in turn below.

One possibility is that the function of interest–differentiation of the words from the pseudo-words–does not happens at the rapid presentation speeds used here. Estimates of word processing time vary widely, reflecting the range of visual, semantic, and attentional processes encompassed by the term. Large-scale data from lexical decision tasks, in which participants typically press a button to indicate whether a rapidly-presented letter string is a word or non-word, suggest response times to words around 700 ms (Keuleers et al., 2012; Yap et al., 2012). This includes the time taken to overtly judge lexicality and press a button to respond. Word processing times in natural reading can be much shorter, falling around 5 Hz, or 200 ms per word (Rayner et al., 2012). Further, when reading, we access the meanings associated with each word and retain them in memory to piece together coherent sentences. However, simply differentiating words from pseudo-words, which this FPVS design targeted, does not *per se* require in depth processing (e.g., full semantic analysis, memory storage, etc.). Semantic access without memory demands can occur with rapid stimulus presentations. Words presented as little as 83 ms apart can elicit an N400, a negative deflection in the ERP typically evoked by a word that is incongruous with its semantic context, even when participants are unable to report them (Luck et al., 1996; see also Rolke et al., 2001). That is, word presentation rates that were too rapid to support attention and recall still elicited a neural response to word meaning. Lexical access would be enough to differentiate our two conditions, and should require less time. This suggests that stimulus onset asynchronies of 100–200 ms in FPVS studies could in theory enable us to quickly measure brain responses that reflect lexical access, despite being faster than natural reading.

On the other hand, lexical decision tasks demonstrate that word/pseudo-word discrimination can be slowed by statistical properties of the stimuli. Decisions are faster for common words (Keuleers et al., 2012; Yap et al., 2012), like those in our study, but can be slower when pseudo-words are built from letter pairs that are common in real words, called “high-frequency bigrams” (Perea et al., 2005; Vergara-Martínez et al., 2013). Our words and pseudo-words were matched on bigram frequency, making them difficult to discriminate. If the lower bound on stimulus presentation times required for lexical access lies between 80 and 200 ms, bigram-frequency-matched pseudo-words could be enough to make our 100 ms stimulus onset asynchronies difficult to follow, contributing to low word detection rates.

Considering how much lower detection rates are in our sample, though, it is surprising that bigram frequency should make such a difference. Lochy et al. (2015) controlled for bigram frequency in a secondary study. They did not report individual effects for this analysis, but reported no change in the group effect, whereas we could not detect an effect in the group. Lochy et al. (2016) further replicated their group effect of periodic word stimulation at a slightly slower pace of 6 Hz base and 1.2 Hz oddball rate. Here we chose 10 Hz base and 2 Hz oddball rate, following Lochy et al. (2015), as this study found the higher detection rate. We were also concerned that a signal below 2 Hz would be difficult to distinguish from low-frequency noise. However, it is promising that Quek and Peelen (2020) recently showed reliable differentiation of object pairs with a 0.625 Hz oddball frequency, albeit only at the group level. It may also be possible to overcome the impact of low-frequency noise by including more trials, without nearing the duration of typical EEG experiments. In the future, it would be interesting to implement these methods using a range of inter-stimulus intervals to test whether oddball frequency responses emerge more reliably for words when base presentation rates approach natural reading speeds (1–2 Hz).

The second concern for the FPVS approach is that word/pseudo-word discrimination may primarily influence ERP components whose latency varies over trials. Whereas differentiation of faces and non-face objects is reliably linked to an early ERP component around 170 ms, there is ongoing discussion about what aspects of word stimuli elicit early (N170) vs. late (N400) variations in the evoked response (see for example Laszlo and Federmeier, 2014). For example, while Hauk et al. (2006) showed divergence in the evoked response to words and bigram-frequency-matched pseudo-words from 160 ms, Vergara-Martínez et al. (2020) emphasised the visual nature of initial word processing by distinguishing early effects of letter case (N150), from late effects of word frequency (N400). This distinction between visual and lexico-semantic processing is mirrored in the organization of the ventral stream. Field potentials recorded from within posterior regions of the human fusiform gyrus that are sensitive to letter strings do not exhibit a preference for words over pseudo-words, whereas recordings in anterior fusiform gyrus do (Nobre et al., 1994).

Critically, late responses may have more variable timing and be less suitable to FPVS entrainment. Recent work highlights between-subject lexical processing variability as key challenge for robust detection. Two studies (Petit et al., 2020a,b) used spoken-word stimuli in a traditional N400 ERP design to measure receptive language in children. Responses in individual children varied in their time-course and topography. If word/pseudo-word discrimination primarily emerges late in the evoked response, and if N400 latencies also vary within individuals, this could undermine the sensitivity of a frequency-specific analysis like FPVS.

Promisingly, time-unconstrained multivariate decoding–which allows detection of effects with different topology and timecourse in each subject–elicited a higher detection rate (16/18 subjects) than traditional ERP analyses in this N400 study (9/18 subjects; Petit et al., 2020a). Such multivariate approaches can theoretically increase sensitivity to time- or location-varying effects by allowing a classifier to learn which channels and timepoints best differentiate experimental conditions in each subject, without increasing multiple comparisons. An exciting new avenue for development uses an updated FPVS design in which stimulation rates change over the course of each trial (Quek et al., 2020). This flexible approach could accommodate uncertainty about the timing of word/pseudo-word differentiation in individuals, by rapidly testing a range of inter-stimulus intervals.

Word-specific responses, where they are seen, seem likely to reflect a cascade of processes, from visual feature segmentation to attention, which we did not attempt to separate here. Clarifying how different aspects of the design (e.g., whether words and pseudo-words have similar or different statistical features, whether participants are asked to attend to the stimuli, whether the timing of oddballs is predictable, etc.; Quek and Rossion, 2017) relate to detection rates will be important for understanding how FPVS can best be used.

In conclusion, fast, periodic stimulation promises rapid insight into mental processes with strong signal relative to noise. Despite many examples of individual-level detection of face processing with FPVS, and preliminary evidence that FPVS can elicit word-specific responses in single subjects, here we showed that individual-level word detection is difficult to probe with FPVS. We suggest that FPVS needs to be developed further before it can serve as a single-subject measure of written word processing.

## Data Availability Statement

The datasets presented in this study can be found in online repositories. The names of the repository/repositories and accession number(s) can be found below: https://osf.io/hkz75.

## Ethics Statement

The studies involving human participants were reviewed and approved by the Macquarie University Human Research Ethics Committee. The patients/participants provided their written informed consent to participate in this study.

## Author Contributions

AW and NB devised the idea, obtained funding, and supervised the project. AW, NB, SP, and LB developed the design. CW, SP, and LB collected the data. SP and LB conducted the analyses. LB wrote the manuscript. All authors discussed the results and contributed to the final manuscript.

## Conflict of Interest

The authors declare that the research was conducted in the absence of any commercial or financial relationships that could be construed as a potential conflict of interest.

## Footnotes

1 https://face-categorization-lab.webnode.com/resources/natural-face-stimuli/

2 http://www.oxfordwordlist.com/

3 http://www.nocions.org/letswave/
