# Supplementary material for: Word Detection in Individual Subjects Is Difficult to Probe With Fast Periodic Visual Stimulation
Source: Front Neurosci. 2021 Mar 3;15:602798. doi: 10.3389/fnins.2021.602798 (PMC7982886; doi:10.3389/fnins.2021.602798)
Supplement: Supplementary file 1 [file Table_1.DOCX]

Supplementary Material

# Word and pseudo-word lists.

**Table S1.** The first letter of each word was changed four times to create four pseudo-words. Each pseudo-word list was then compared to the word list for bigram frequency.

| *Words* | *Pseudo-words* | | | |
| --- | --- | --- | --- | --- |
|  | *1* | *2* | *3* | *4* |
| Back  Book  Call  Case  Down  Feet  Five  Food  Four  Girl  Hand  Head  Home  Land  Line  Love  Name  Poor  Road  Room  Talk  Time  Wife  Work | Vack  Wook  Vall  Gase  Fown  Keet  Bive  Nood  Bour  Dirl  Dand  Gead  Fome  Pand  Gine  Nove  Bame  Loor  Moad  Foom  Dalk  Nime  Tife  Nork | Gack  Dook  Nall  Nase  Rown  Peet  Mive  Tood  Kour  Firl  Tand  Tead  Gome  Gand  Hine  Kove  Kame  Noor  Foad  Goom  Ralk  Pime  Mife  Mork | Nack  Gook  Jall  Pase  Cown  Leet  Kive  Sood  Wour  Kirl  Jand  Kead  Lome  Cand  Rine  Bove  Rame  Soor  Joad  Toom  Nalk  Fime  Dife  Lork | Cack  Mook  Kall  Hase  Pown  Deet  Tive  Vood  Nour  Lirl  Mand  Sead  Pome  Vand  Rine^1^  Sove  Vame  Coor  Noad  Soom  Salk  Sime  Pife  Bork |

^1^Due to an oversight, ‘rine’ appeared in pseudo-word lists 3 and 4

# Word-specific response detection rates adjusted for multiple ROIs and stimulus sets.

We tested whether people showed a word-specific response in four different ways: with two stimulus sizes, in two regions of interest. We did this to sensitively test whether we can elicit word-specific responses in a high proportion of people. However, for transparency, here we show the detection rates after correction for the number of tests we conducted.

First, we show the number of individuals who met the standard criteria for a word-specific response. We break it down by condition to show how many would have met those criteria had we limited our study to one ROI and one stimulus set. For the most successful combination, if we were to use only large stimuli and exclusively looked at the left ROI (the typical site of language lateralisation), our detection rate would be 2 out of 10 people (20%).

Second, we recompute the z-scores we used to judge detection rates. The original method asks whether the frequency of interest (2 Hz and its harmonics) is larger than the 20 surrounding frequency bins. This should only happen by chance 5% of the time. We reran this analysis, selecting the surrounding 40 and 80 bins to establish 2.5% and 1.25% false positive rates. This corrects for two (stimulus sets) or four (ROIs*stimulus sets) comparisons. The frequency resolution was .0166 Hz, meaning that 80 bins around 2 Hz, excluding the first neighbouring bins, captured the range from 1.30 Hz to 2.70 Hz. When we control the false positive rate at 1.25%, no participant meets the criteria for a word-specific response in any condition.

**Table S2.** Number of individuals (out of ten) who met the criteria for a word specific response in each condition, controlling the false positive rate at 5%, 2.5%, and 1.25%.

| *alpha* | *Small stimuli* | | *Large stimuli* | |
| --- | --- | --- | --- | --- |
|  | *Left ROI* | *Right ROI* | *Left ROI* | *Right ROI* |
| *.05* | 1 | 0 | 2 | 1 |
| *.025* | 1 | 0 | 2 | 1 |
| *.0125* | 0 | 0 | 0 | 0 |

# Individual participant signal-to-noise ratios

**Figure S3.** Individual participant frequency spectra for (A) faces among scrambled faces, (B) words among pseudo-words, and (C) large words among pseudo-words at the two ROIs and Oz. Signal-to-noise (SNR) units represent the ratio of the amplitude at each frequency to the mean amplitude of the 20 neighbouring frequencies, excluding the nearest frequency bin on either side. A new stimulus appeared every 200 ms, eliciting a visual steady state responses at 10 Hz in both left and right ROIs, and at Oz. For all conditions, the group-level base stimulation response was strongest at Oz. Face or word oddballs appeared every 500 ms. Oddball-specific responses were expected at 2 Hz and its harmonics (4 Hz, 6 Hz, 8 Hz). This was observed at the group level in the face condition, but in neither word condition.

1. **Faces**


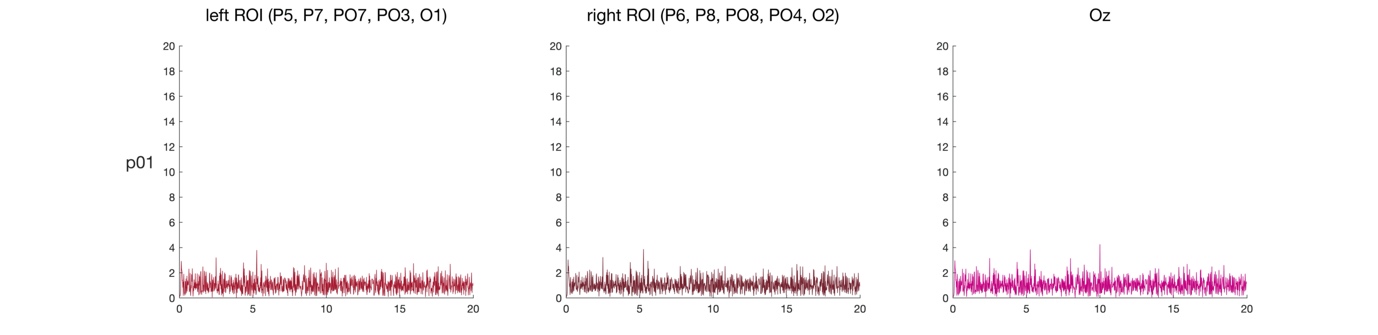

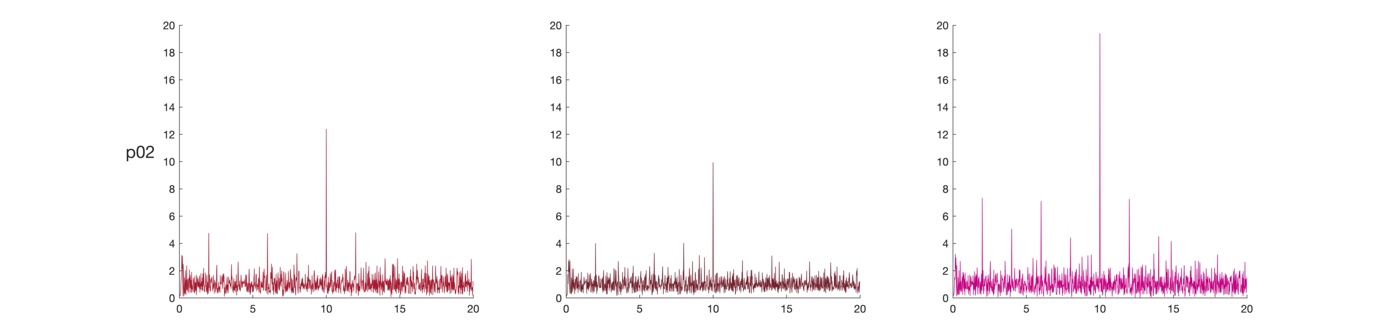

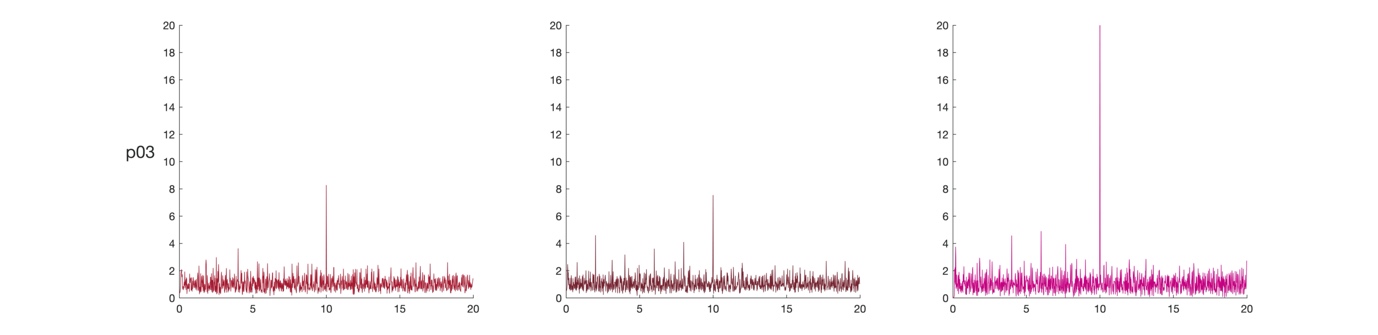

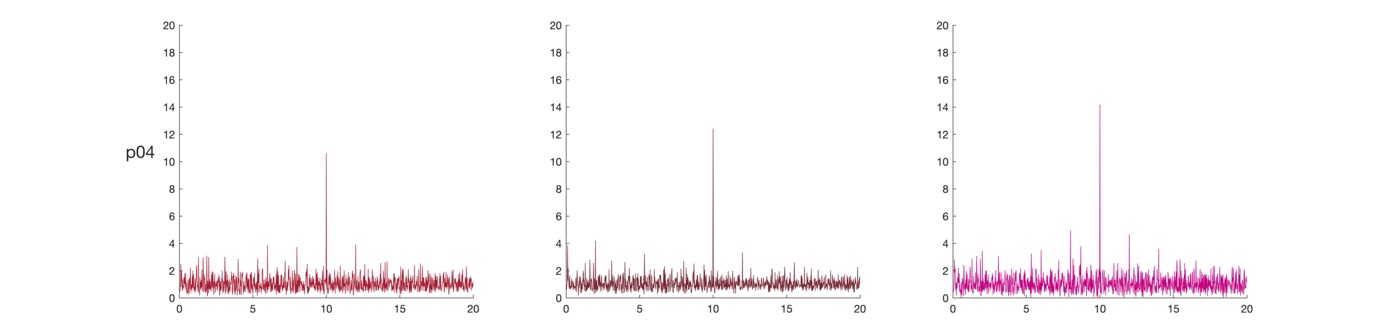

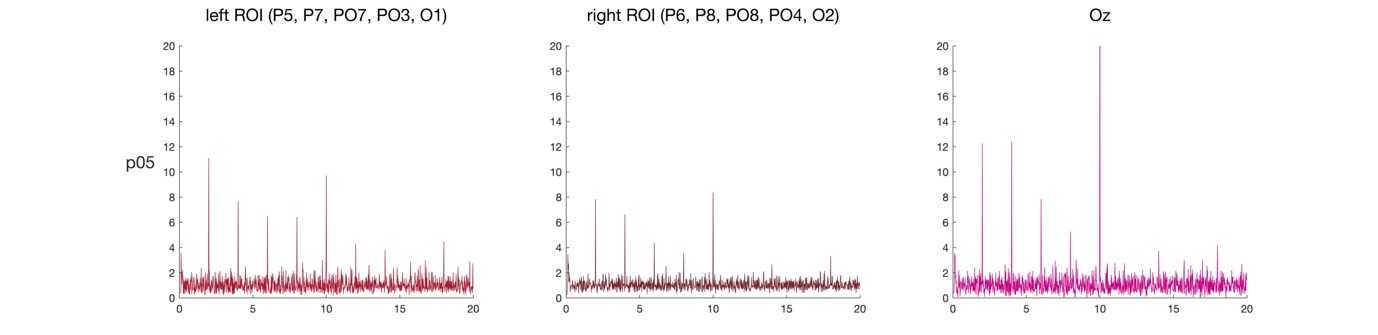

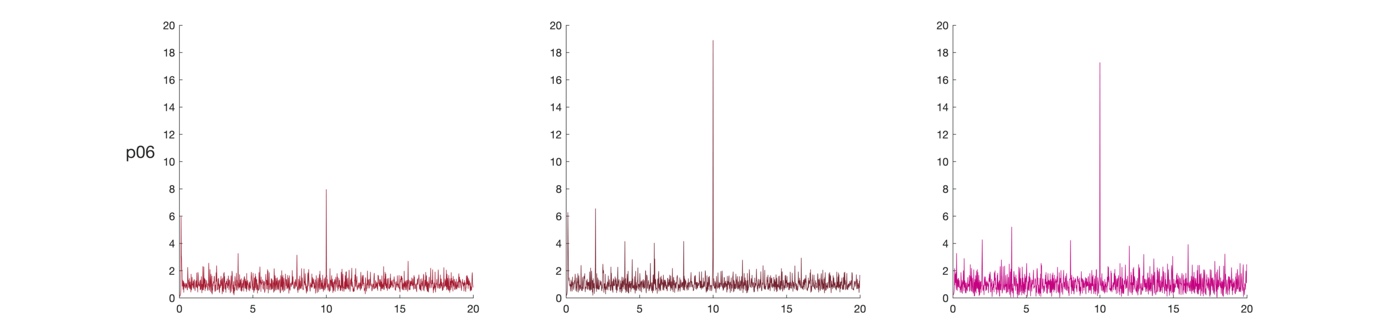

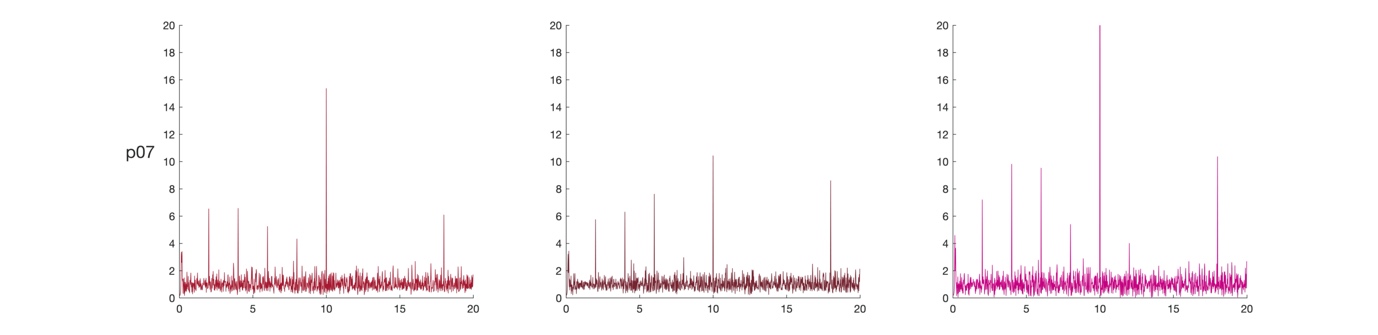

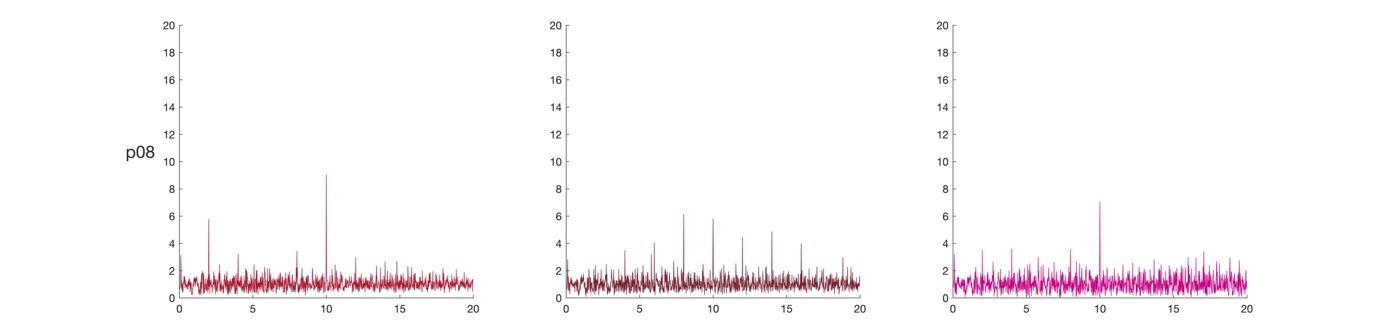

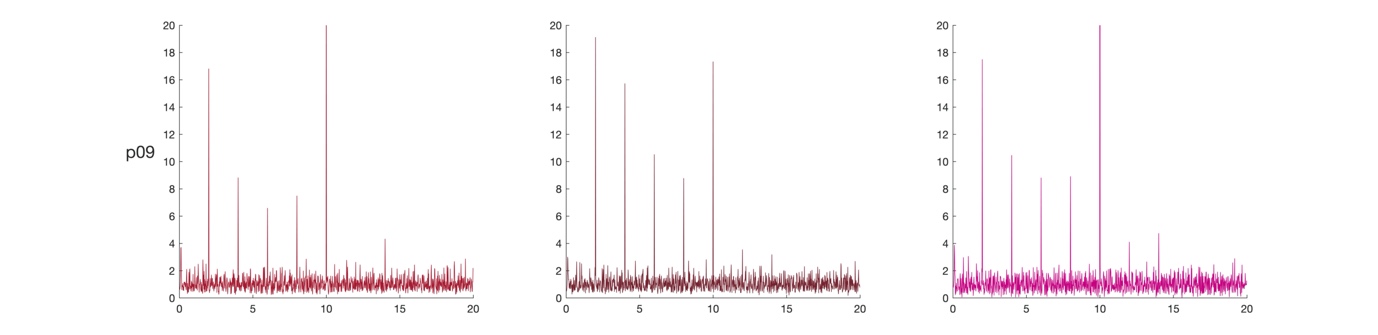

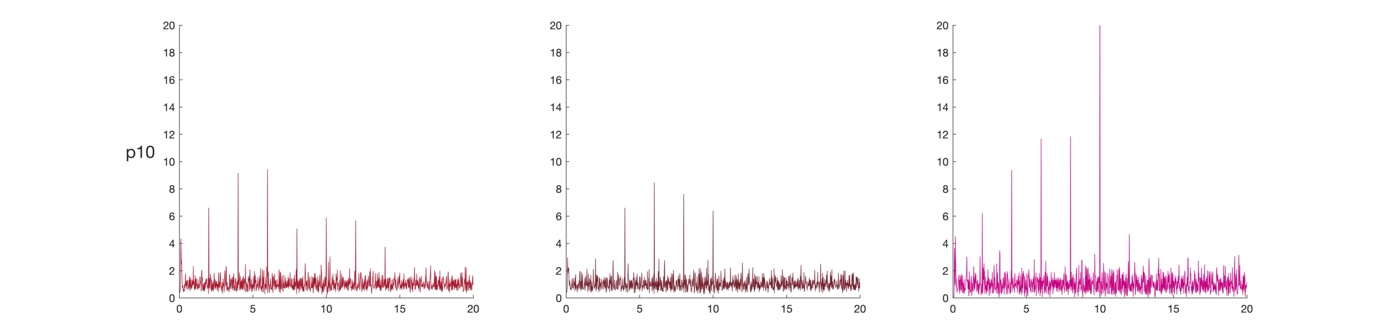


SNR

Frequency (Hz)

SNR

Frequency (Hz)

1. **Words (original size)**

**
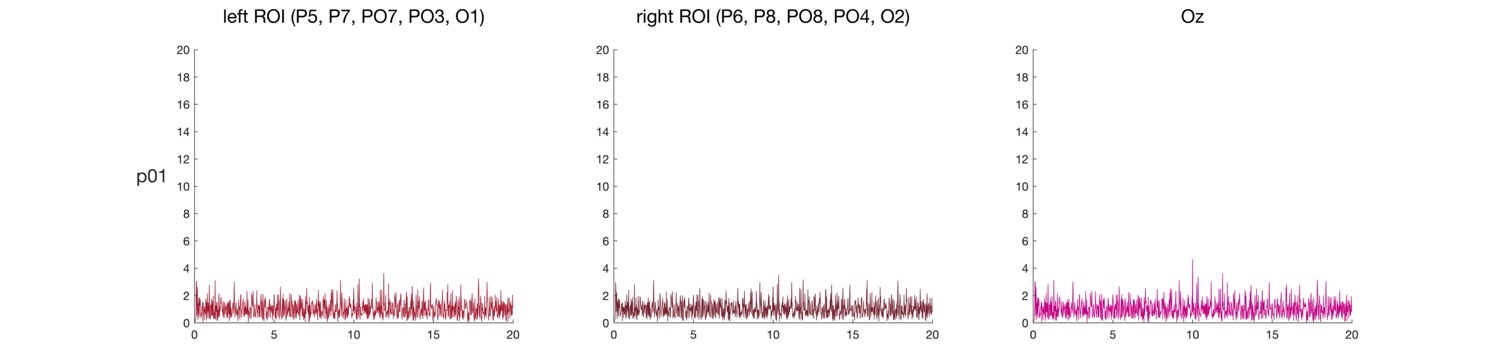

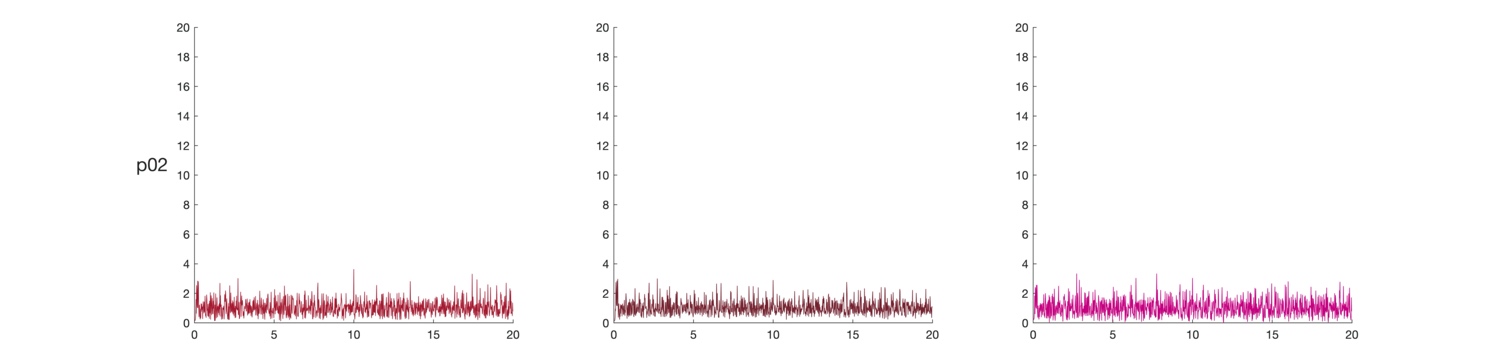

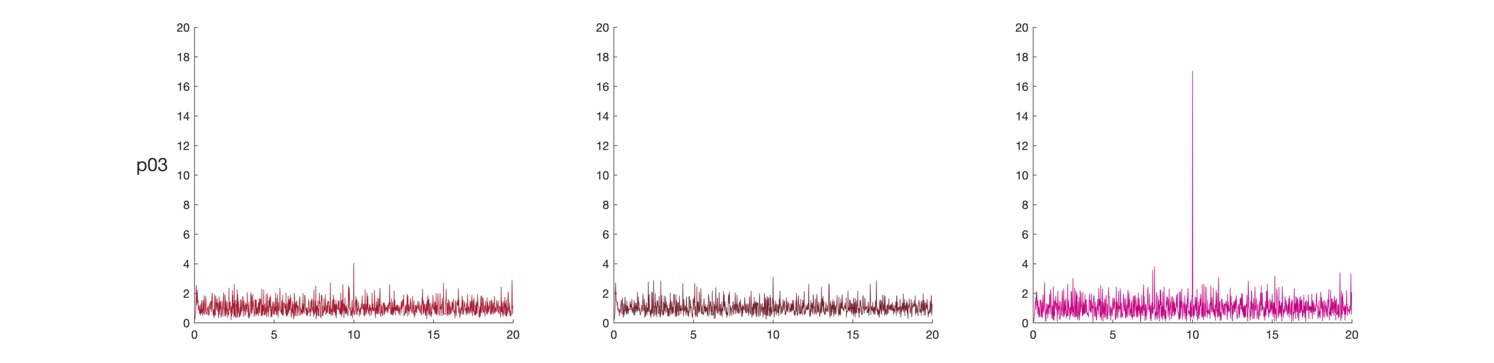

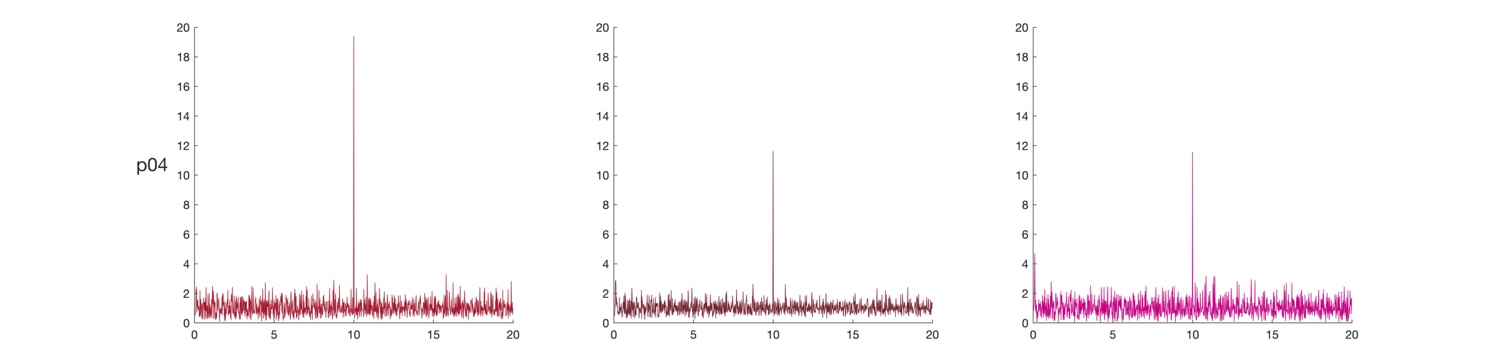

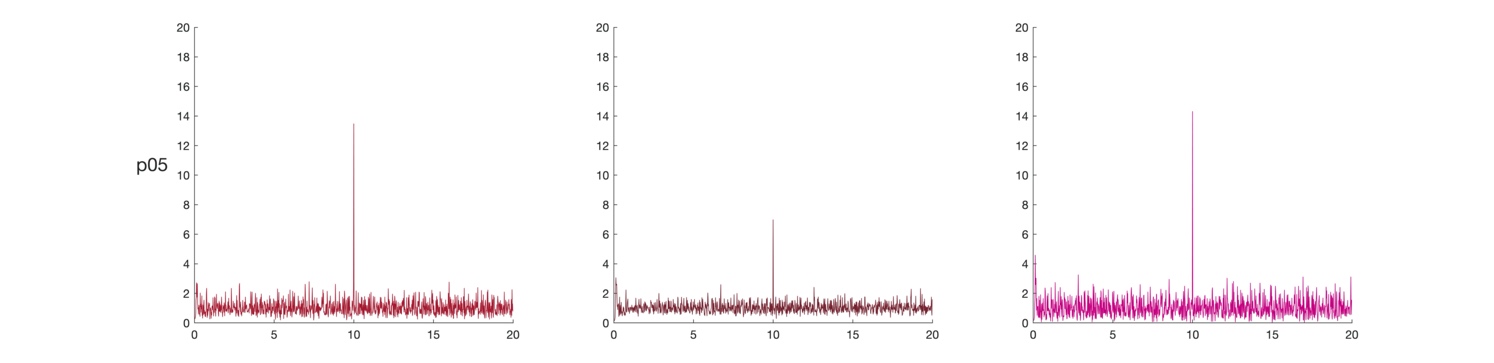

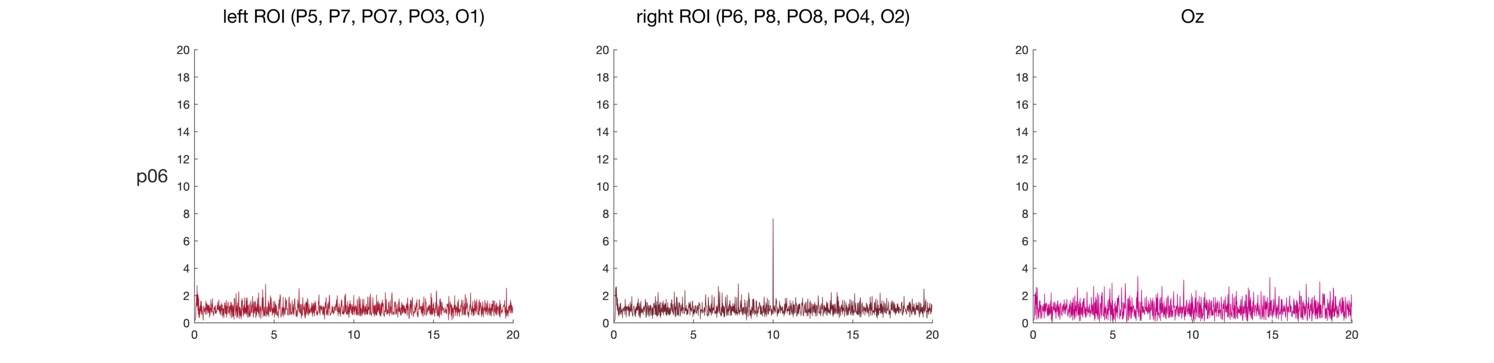

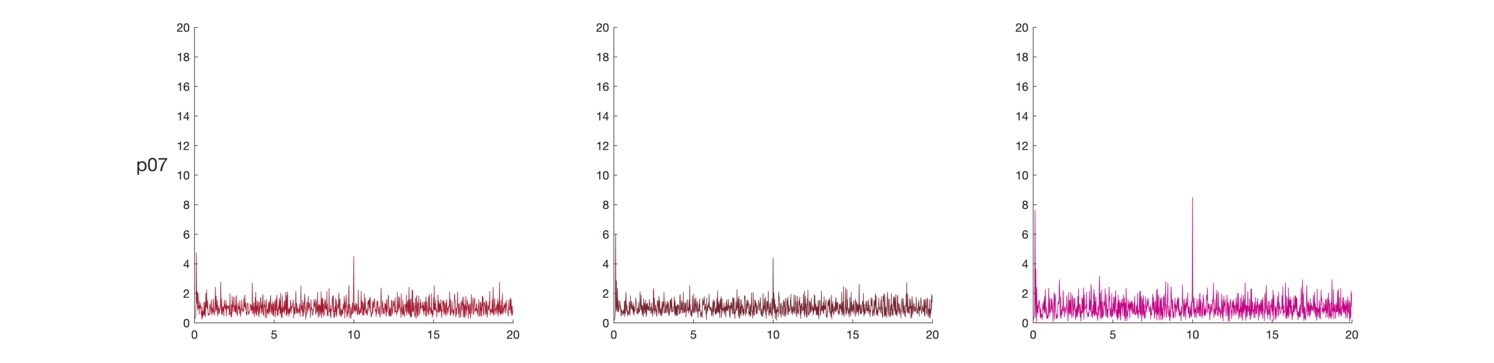

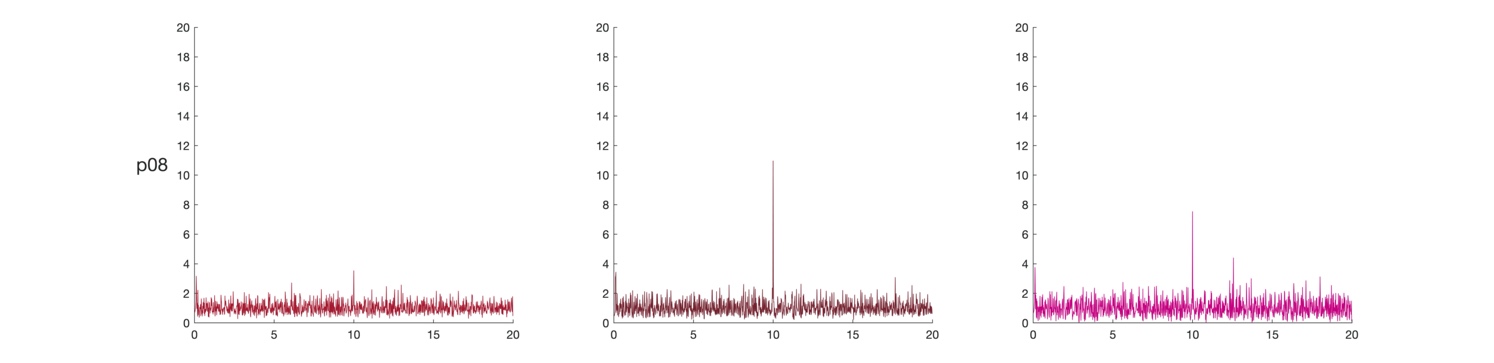

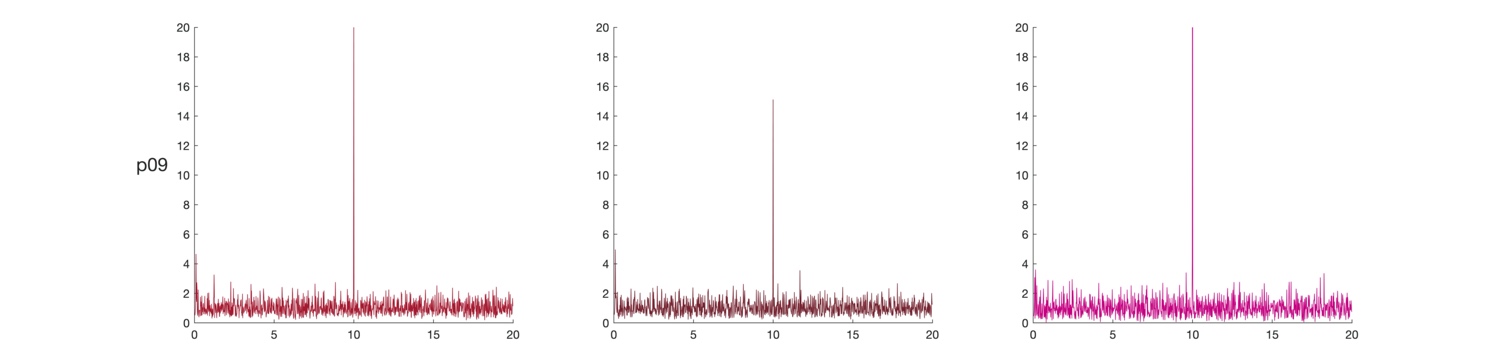

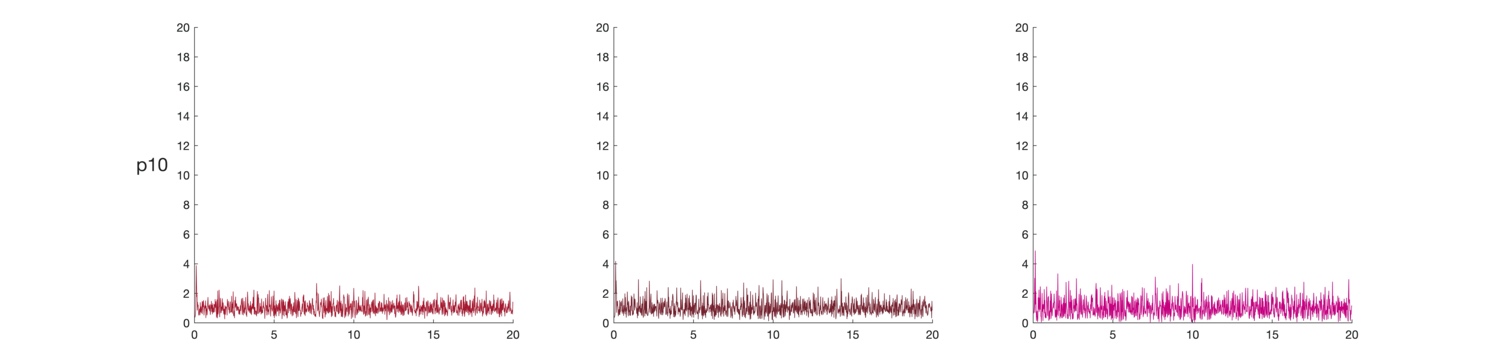
**

SNR

Frequency (Hz)

SNR

Frequency (Hz)

SNR

Frequency (Hz)

1. **Words (large)**

**
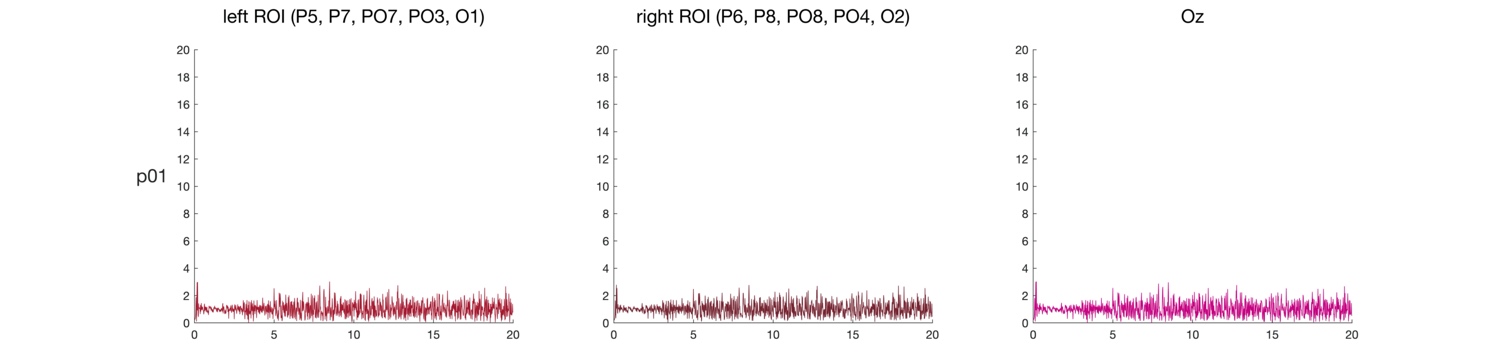
**

**
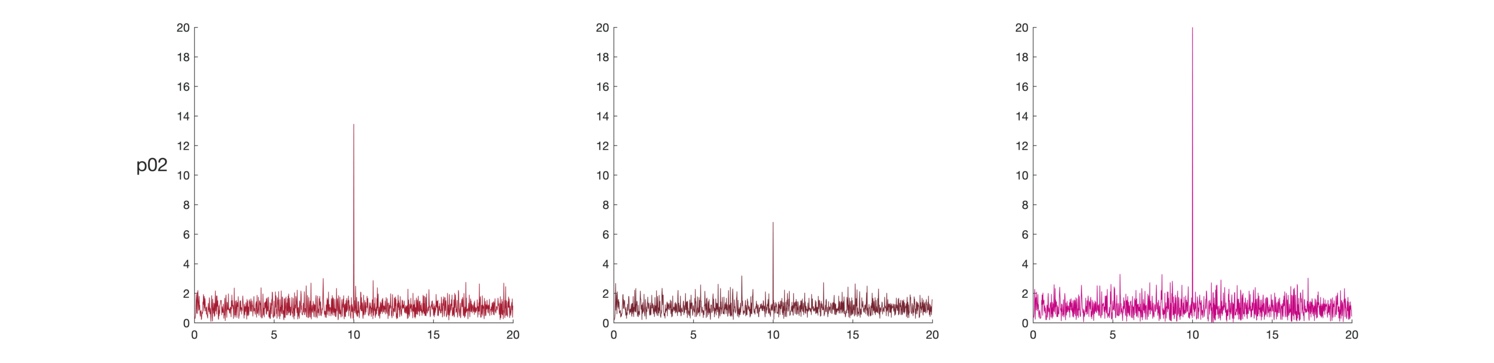

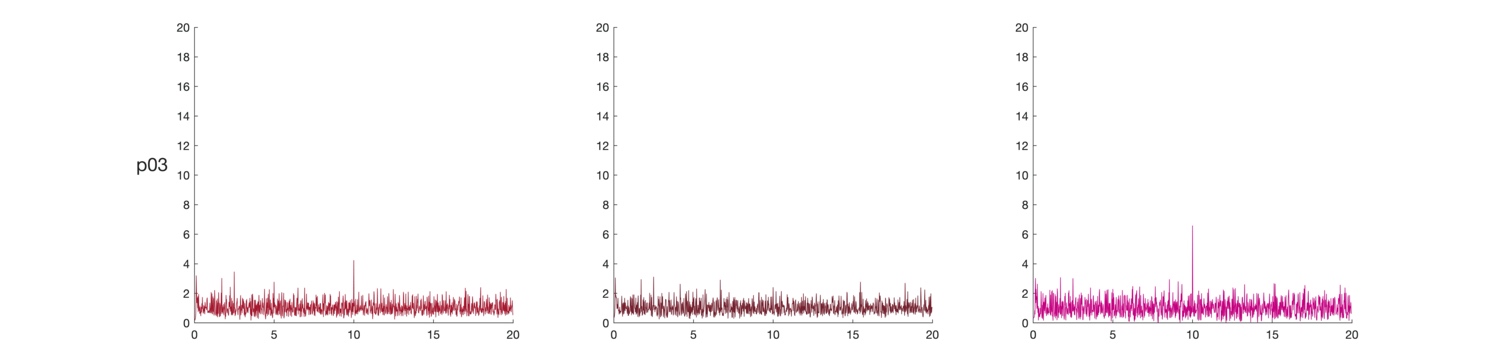

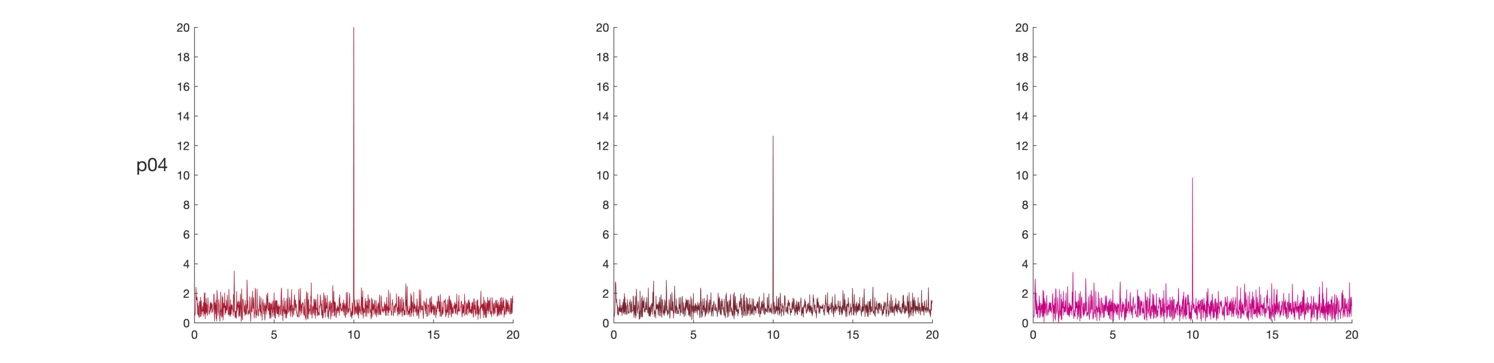

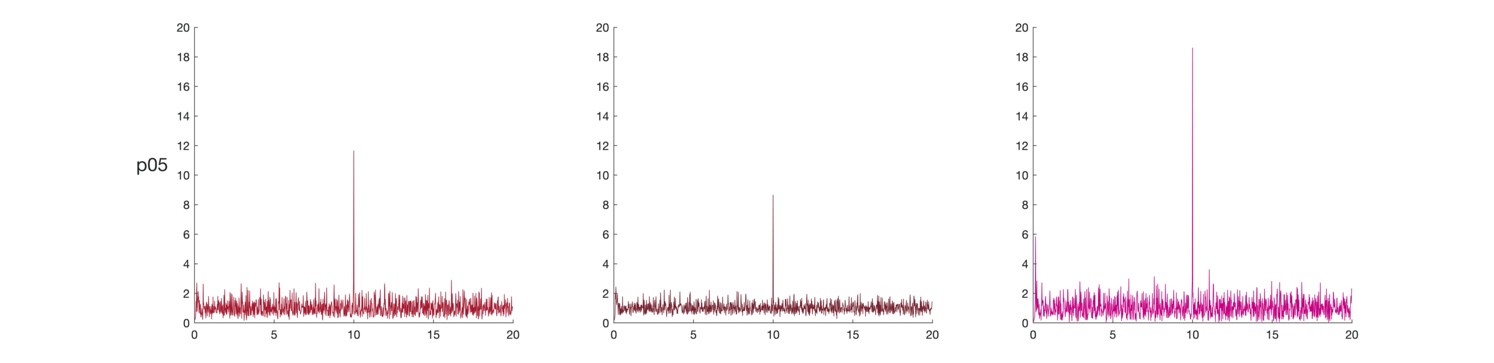

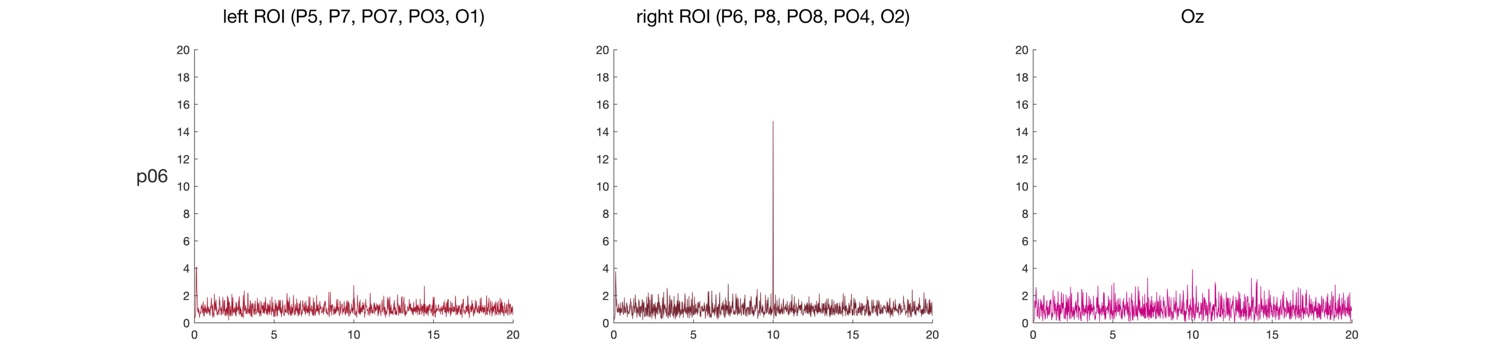

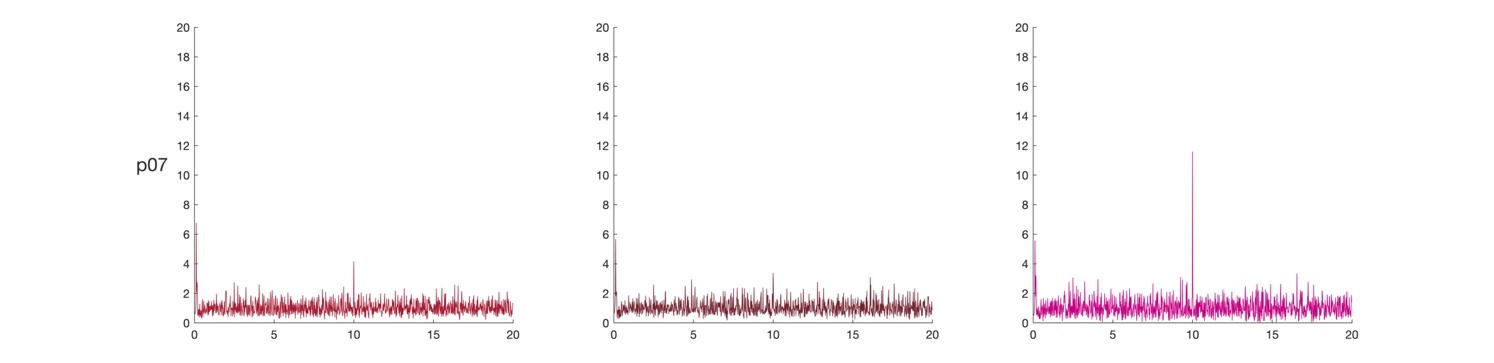

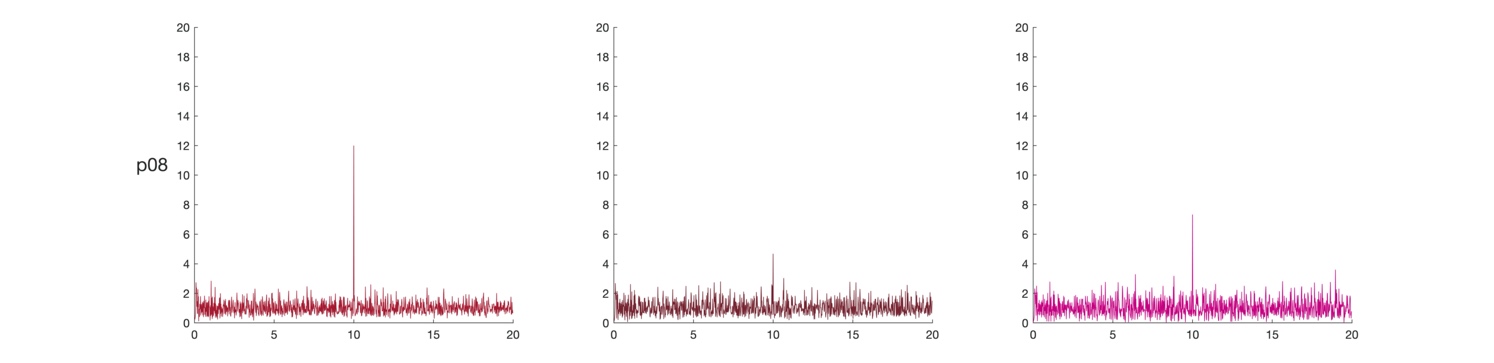

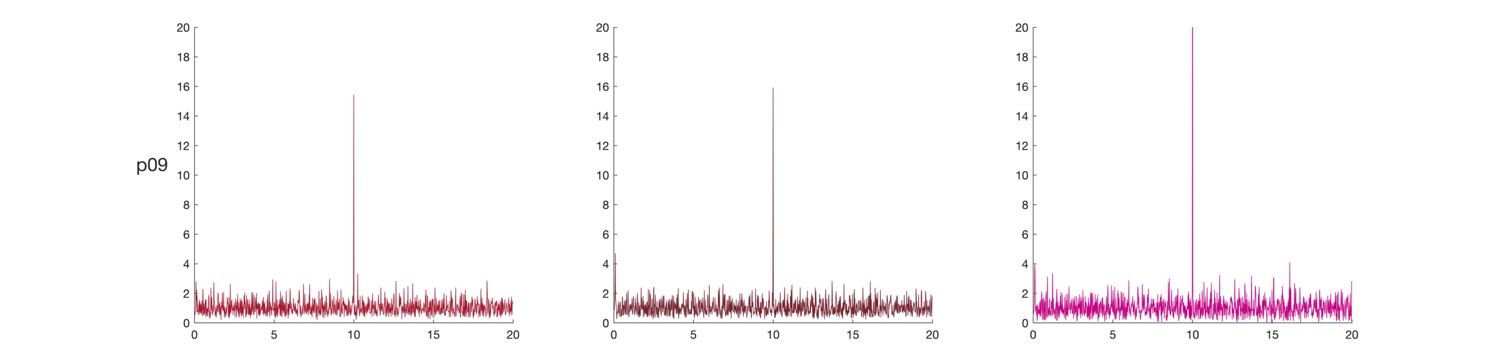

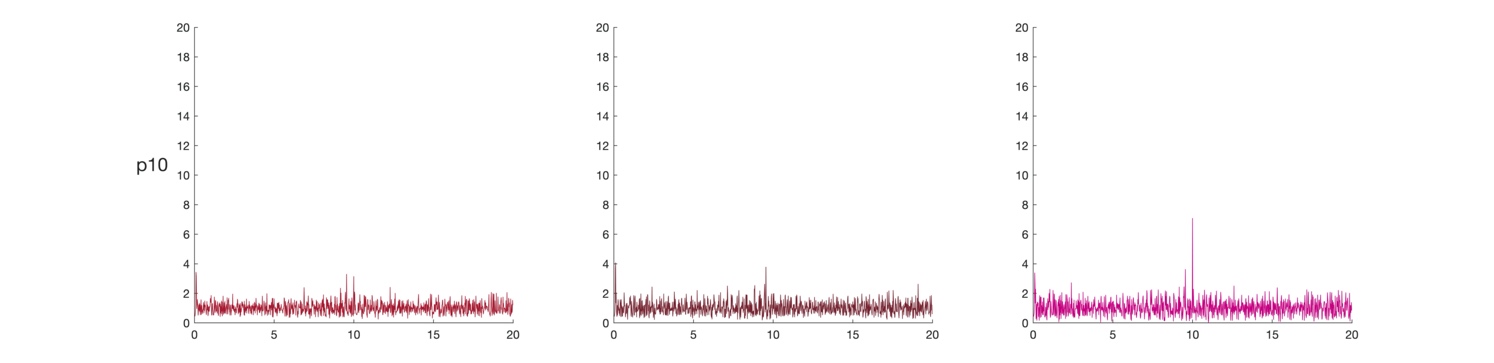
**

SNR

Frequency (Hz)

SNR

Frequency (Hz)
